# There is no health without mental health: Challenges ignored and lessons learned

**DOI:** 10.1002/ctm2.897

**Published:** 2022-05-31

**Authors:** Alvina G. Lai, Wai Hoong Chang

**Affiliations:** ^1^ Institute of Health Informatics University College London London UK

**Keywords:** Mental health in children, mental health, physical comorbidities

In his investigations of the relationship between the mind and the body, René Descartes, the father of modern philosophy, coined the term Cartesian dualism, colloquially known as mind–body dualism. According to Descartes, the mind and body are ontologically distinct entities. Later, a philosopher and psychiatrist, Karl Jaspers, claimed that psychological and biological investigations of the mind were akin to "*the exploration of an unknown continent from opposite directions"*. Quoting Jaspers, Gardner and Kleinman note that limitations in the biological understanding of disorders of the mind have continued to plague the field of Psychiatry, leading to an identity crisis that calls for a rethinking of how practitioners diagnose and treat mental illness.^1^

The Cartesian dualism is thought to be too restrictive and could undermine the mental struggles of patients. Numerous studies have contested this metaphysical stance. In a study examining 459,542 patients with cancer, we found that certain cancer types and cancer treatments were associated with a higher burden of mental illness, suggesting that physical conditions can exert variable impacts on mental health.^2^ Our analyses also demonstrated that patients with cancer and mental illness were more likely to harm themselves, and the risk of self‐harm was significantly higher within the first 12 months of a mental illness diagnosis. Our study cast a spotlight on the complex interaction between mental and physical morbidities that could result in lasting health outcomes if left unaddressed.

Greater awareness of the relationship between psychiatric disorders and physical conditions would not only have profound implications on the mental and physical well‐being of real patients but also for clinical practice in general. Yet, for many years, there remains a substantial unmet need for addressing the multifaceted nature of psychiatric disorders. The Diagnostic and Statistical Manual of Mental Disorders (DSM‐5) uses checklists of diagnostic criteria; however, the practice of diagnostic assessment is thought to be contentious.^3^ There is considerable heterogeneity within the diagnostic criteria—a study found that for the majority of diagnoses in DSM‐5 and DSM‐IV‐TR, two individuals could receive the same diagnosis without sharing any symptoms.^4^

While DSM‐5′s model of discrete disorders offers much clinical utility by providing a definitive list of symptoms, there is more to be done to incorporate individualised adjustments to account for heterogeneity and symptom overlap between diagnoses. The focus on broad diagnostic categories and one‐size‐fits‐all approaches has largely disregarded other factors (i.e., cultural practices, interpersonal relationships, socioeconomic statuses and the presence of comorbid physical conditions). Therapies and medications are mainly prescribed based on diagnoses^5^ with limited consideration of extraneous symptoms.

Mental health remains a largely neglected global issue despite being critical to the attainment of the United Nations 2030 Sustainable Development Goals. The coronavirus pandemic has taught us that mental health is profoundly affected by the circumstances in which we live. Even as the virus becomes less deadly, the negative social and economic effects could create lasting damages that disproportionately affect vulnerable communities. Children with chronic physical conditions are more susceptible to mental disorders.^6^ Globally, 25% of children live with a chronic physical condition and children with mental–physical comorbidities experience higher impairments in both physical and mental health.^7^

For mental health interventions to be effective, we need to examine the interactions between physical and psychological health and social factors, as informed by the lived experience of real people. In response to the limited improvements in mental health outcomes over the last few decades, Wellcome identified and reviewed 46 active ingredients, which are aspects of mental health intervention that drive clinical effect. These active ingredients can be divided into six groups: (1) behaviours and activities, (2) beliefs and knowledge, (3) brain and body functions, (4) cognitive and attentional skills, (5) human connections, and (6) socioeconomic factors.^8^

Mental health is linked to critical periods of brain development and 75% of mental health problems in adulthood start before age 18.^9^ Early intervention is a key, however, relatively little is known about the efficacy and safety of medications for mental illness in children. Off‐label prescribing of psychotropic medications is common in children, but off‐label use of medicines may increase the risk of adverse side effects.^10^, ^11^ Children are receiving powerful medications that have only been tested for use in adults. A systematic review of 80 psychotropic medications and 78 adverse events found safer profiles for certain medications used in children.^12^ Results from this systematic review should be interpreted with caution because data for adverse events are limited for many of the reviewed medications, and information is based on spontaneous reports which may underestimate the actual frequency. The study only focuses on adverse events and did not explore how effective each medication is in children. The question of medication safety becomes more pressing in children with comorbid physical conditions. Polypharmacy is more likely in these children and the field has yet to release a comprehensive guide for treating mental illness in the presence of other conditions.

While targeted clinical care focusing on psychiatric conditions alone might provide a streamlined solution for dealing with increasing demands in an era of overstretched systems and conveyor belt medicine,^13^ this oversimplified design often ignores the wider influences of other domains. Individuals with physical conditions may face competing health and care demands for both mental and physical disorders. Psychotropic drugs may interact with medications used to treat other conditions such as cancer^14^ and epilepsy.^15^ Additionally, our study on patients with cancer found that individuals with a higher number of comorbidities were less likely to receive a diagnosis of mental illness before their first self‐harm event.^2^ This suggests that morbidity burden has an impact on help‐seeking behaviour before self‐harm. We also found that patients from more socioeconomically deprived communities were less likely to receive a diagnosis of mental illness before their first self‐harm episode, which highlights the importance of community‐level efforts to support access to care (see Figure 1).

**FIGURE 1:**
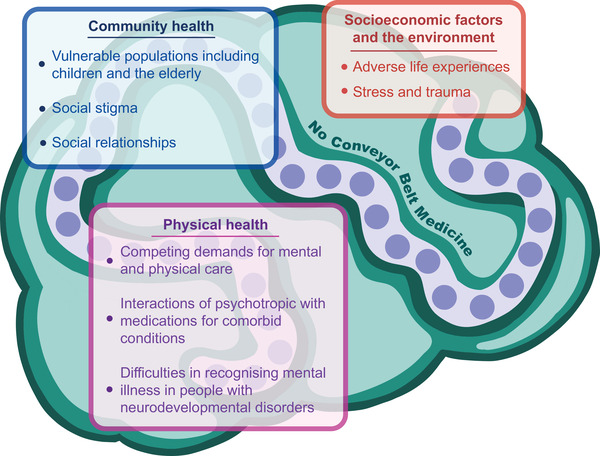
Critical determinants of mental health highlighting the interdependence of social, community and physical domains. The heterogeneity of mental illness suggests that a conveyor belt approach to diagnosis and treatment is insufficient. People with chronic physical conditions require individualised adjustments, drawing knowledge from multiple clinical specialties including community studies to improve the overall quality of care.

Diagnosing and treating mental illness in people with neurodevelopmental disorders (i.e., autism spectrum disorder) become infinitely more complex.^16^ Existing diagnostic guidelines for anxiety and depression focus on conversational techniques^5^ without accounting for speech and language difficulties in people with autism. A high proportion of children with autism present with comorbid symptoms of attention deficit hyperactivity disorder (ADHD) and psychostimulants used to treat ADHD may interact with antidepressants.

Much work is needed to better understand, diagnose and treat mental disorders by drawing in expertise from psychiatry, neurology and other clinical specialties that manage physical conditions. We need to bridge clinical silos and move beyond the single‐disease model to provide better mental health care. We need to focus on whole‐person care by engaging in open conversations with patients to consider their priorities. Expanding the field to include social health and the environment may reveal crucial aetiological factors of mental illness. Without recognising these interrelated factors, Occam's razor, also known as the principle of parsimony seeking to provide the simplest explanation for a problem, rapidly loses its value in responding to the mental health needs of individuals and communities.

## CONFLICT OF INTEREST

The authors declare that there is no conflict of interest that could be perceived as prejudicing the impartiality of the research reported.
